# Face Perception and Pareidolia Production in Patients With Parkinson's Disease

**DOI:** 10.3389/fneur.2021.669691

**Published:** 2021-08-03

**Authors:** Nicole Göbel, Jens Carsten Möller, Nathalie Hollenstein, Andreas Binder, Matthias Oechsner, Jörg Ide, Prabitha Urwyler, Dario Cazzoli, René M. Müri

**Affiliations:** ^1^Perception and Eye Movement Laboratory, Departments of Neurology and BioMedical Research, Inselspital, Bern University Hospital, and University of Bern, Bern, Switzerland; ^2^Department of Neurology, Inselspital, Bern University Hospital, Bern, Switzerland; ^3^Rehaklinik Zihlschlacht, Centre for Neurological Rehabilitation, Zihlschlacht-Sitterdorf, Switzerland; ^4^Department of Neurology, Philipps University, Marburg, Germany; ^5^Gerontechnology and Rehabilitation Group, ARTORG Center, University of Bern, Bern, Switzerland

**Keywords:** Parkinson's disease, non-motor symptoms, face perception, misperception, pareidolia, hallucination, embedded face paradigm

## Abstract

In Parkinson's disease (PD) patients, visual misperceptions are a major problem within the non-motor symptoms. Pareidolia, i.e., the tendency to perceive a specific, meaningful image in an ambiguous visual pattern, is a phenomenon that occurs also in healthy subjects. Literature suggests that the perception of face pareidolia may be increased in patients with neurodegenerative diseases. We aimed to examine, within the same experiment, face perception and the production of face pareidolia in PD patients and healthy controls (HC). Thirty participants (15 PD patients and 15 HC) were presented with 47 naturalistic photographs in which faces were embedded or not. The likelihood to perceive the embedded faces was modified by manipulating their transparency. Participants were asked to decide for each photograph whether a face was embedded or not. We found that PD patients were significantly less likely to recognize embedded faces than controls. However, PD patients also perceived faces significantly more often in locations where none were actually present than controls. Linear regression analyses showed that gender, age, hallucinations, and Multiple-Choice Vocabulary Intelligence Test (MWT) score were significant predictors of face pareidolia production in PD patients. Montreal Cognitive Assessment (MoCA) was a significant predictor for pareidolia production in PD patients in trials in which a face was embedded in another region [*F*_(1, 13)_ = 24.4, *p* = <0.001]. We conclude that our new embedded faces paradigm is a useful tool to distinguish face perception performance between HC and PD patients. Furthermore, we speculate that our results observed in PD patients rely on disturbed interactions between the Dorsal (DAN) and Ventral Attention Networks (VAN). In photographs in which a face is present, the VAN may detect this as a behaviourally relevant stimulus. However, due to the deficient communication with the DAN in PD patients, the DAN would not direct attention to the correct location, identifying a face at a location where actually none is present.

## Introduction

Illusions (misperceptions of real stimuli) and hallucinations (aberrant perceptions) are a major problem within non-motor symptoms of Parkinson's disease (PD). They occur in more than half of all patients with advanced disease (1–5). In early disease, older patients with slight decline of cognitive function were at higher risk of developing hallucinations under treatment (6). Furthermore, in 35-40% of drug-naive “*de novo*” PD patients, minor hallucinations were reported (7) with gray matter loss in MRI being a risk factor (8). Flickering, false impressions, and illusionary misperceptions precede the core syndrome of stereotyped, colorful hallucinatory images (9). In visual hallucinations and illusions, most common “people” are reported (10). Often, PD patients realize that they are hallucinating, though patients with dementia may lose this insight (3).

Psychosis is commonly associated with dementia (1). The pathophysiology underlying visual misperceptions and hallucinations in PD is not well-understood. It has been suggested that pathophysiological changes in PD result in altered cortical visual processing. Stebbins and colleagues (11) compared cerebral activation patterns in PD patients with and without visual hallucinations using fMRI. They found that PD patients with hallucinations respond to visual stimuli with greater frontal and subcortical activations, and less visual cortical activations, than non-hallucinating subjects. Meppelink and colleagues (12) examined the cerebral activation patterns using fMRI and found bilateral activations of the fusiform and lingual gyri during image recognition, however, patients with hallucinations additionally showed a reduced activation of the lateral occipital cortex and of extrastriate temporal visual cortices, several seconds before image recognition. Thus, impaired visual object processing in occipital and temporal extrastriate visual cortices supports the hypothesis of impaired bottom-up visual processing in PD with hallucinations (12).

A number of researchers have suggested a key role of perceptual and attentional deficits in the development of visual hallucinations (12, 13). Ramirez-Ruiz and colleagues (14) suggested a model of hallucinations that is based on a relative inability of the brain to recruit the dorsal attention network in the presence of an ambiguous percept. The dorsal attention network is thought to be critical for directing attention and encoding neural signals related to the behavioral significance of a stimulus (14). Distribution of gray matter atrophy suggests that visual hallucinations are linked to aberrant activity within visual thalamo-cortical networks (15).

In this context, the phenomenon of pareidolia is of interest. Pareidolia is an illusory sensory perception that is common in healthy subjects. It has been suggested that pareidolia occurs if external visual stimuli (such, e.g., cloud shapes) trigger a perception of a (non-existing) entity. Pareidolia may represent erroneous matches between internal representations and the sensory input (16). As such, pareidolia represent a possibility to study visual integration of bottom-up and top-down information.

Among all forms of pareidolia, face pareidolia are well-documented and described since Leonardo da Vinci (17). The phenomenon of face pareidolia suggests that the visual system is highly tuned to perceive faces, likely due to their social importance and our exquisite ability to process them. Perhaps because of a side effect of quick face perception, we find faces in inanimate objects such as in the front view of cars and trains and in spots on walls and ceilings (18).

Several studies examined the occurrence of pareidolia in neurological and psychiatric diseases (19–21). Uchiyama and colleagues (19) used a newly developed test for evoking pareidolic illusions in patients with dementia with Lewy bodies (DLB), in patients with Alzheimer's disease, and in healthy controls. They found that, in DLB patients, a much greater number of pareidolia were produced compared to Alzheimer's disease patients and to controls. In another study (20), examined pareidolia production in patients with Parkinson Disease (PD) without dementia and in a control group, investigating brain activity by means of 18F-fluorodeoxyglucose positron emission tomography (FDG-PET). PD patients produced more pareidolia than controls, and the number of pareidolia correlated with hypometabolism in the temporal, parietal, and occipital cortices of both hemispheres. The occurrence of visual hallucinations was correlated with hypometabolism in the left parietal cortex. They concluded that posterior cortical dysfunction could be a common neural mechanism of pareidolia, and that pareidolia could represent subclinical hallucinations or a predisposition to visual hallucinations. Murakami and colleagues (22), using SPECT in drug-naive PD patients, showed that the perception of face pareidolia is associated with significantly lower uptake of l-ioflupane in the right striatum in patients with than without pareidolia.

The aim of our study was to examine face perception and the production of face pareidolia in PD patients, and to compare their performance with age-matched healthy controls (HC). The literature shows that the ability to recognize faces is impaired in patients with Parkinson's disease (23) and that face pareidolia is the most frequent form of pareidolia (19, 24). For this reason, we developed the so-called embedded face paradigm using complex visual naturalistic photographs, in which faces were embedded or not. The likelihood to perceive the embedded faces was modified by manipulating their transparency. The novelty of our paradigm is that, thanks to the embedded faces, face perception and face pareidolia production can be simultaneously evaluated. Furthermore, we believe that the use of non-noise and non-blurred images may more closely reflect everyday situations.

Our hypothesis was that PD patients would show a reduced perception of embedded faces, but an increased number of face pareidolias, compared to the control group. Furthermore, we were interested in assessing whether disease-specific parameters—such as cognitive performance, UPDRS values, or duration of the disease—would be predictive.

## Materials and Methods

### Subjects

Thirty subjects (15 PD patients and 15 HC) were included. The mean age of the 15 PD patients was 65.2 years (SD = 10.8 years, 7 females and 8 males), the mean age of the 15 HC was 64.1 years (SD = 7.7 years, 9 females and 6 males). On average, the PD patients received their clinical PD diagnosis 11.6 years (SD = 6.7 years) before inclusion in the study. Mean years of education were 13.7 years (SD = 3.9 years) in the PD group and 12.9 years (SD = 3.2) in the control group. The mean Montreal Cognitive Assessment (MoCA) score in the PD group was 25.8 (SD = 2.3), in the control group 28.4 (SD = 1.4). Subjects showing a MoCA score <26 were considered as presenting with cognitive impairment. In the PD group, 6 patients showed cognitive impairment, whereas in the control group no subject showed a MoCA below 26. Fourteen PD patients were right-handed, one left-handed. In the control group, fourteen individuals were right-handed, one ambidexter. There was no significant difference between controls and PD patients concerning age (*p* = 0.742), education (*p* = 0.545), gender (*p* = 0.464), or handedness (*p* = 0.962). MoCA scores were significantly different (*p* = 0.002) between PD patients and controls.

All PD patients were treated with Levodopa (mean = 1058 mg, SD = 488.3 mg). Three PD patients were implanted with deep brain stimulators (DBS). Average Hoehn-Yahr (*H-Y*) stage was 3 (SD = 1.0). All PD patients and subjects had a binocular vision of 0.3 or better, and no other neurological, psychiatric, or ophthalmological disease. The Movement Disorder Society-sponsored revision of the Unified Parkinson's Disease Rating Scale (MDS-UPDRS) (25) was used as a clinical assessment tool in PD disease, especially Part 1. The Hoehn & Yahr scale (26) was used to assess the severity of the disease in PD patients. The German version of the North East Visual Hallucinations interview (NEVHI) (27) was used to evaluate visual hallucinations. Stroop errors of the Victoria Stroop Test were used for measuring the individual interference tendency (28). Furthermore, the Multiple-Choice Vocabulary Intelligence Test (“Mehrfachwahl-Wortschatz-Intelligenztest,” MWT) (29) was administered to evaluate general intelligence level. The demographics of the participants are shown in Table 1.

**Table 1:** Participants data.

**Subjects^**a**^**	**Age range**	**Education**	**MoCA^**b**^**	**normalized**	**MWT**	**MoCA**	**Visual**	**MDS UPDRS**	**DBS**	**H-Y**	**Levodopa**
	**(years)**	**(years)**		**Stroop errors**	**score**	**score**	**hallucinations**	**Part 1**		**Stage**	**(mg)**
P1	76–80	10	25^*^	−0.2	111	25	Y	9	no	4	850
P2	71–75	10	29	−0.2	134	29	N	8	no	4	2195
P3	51–55	21	28	−0.8	139	28	Y	10	no	2	1150
P4	66–70	11	26	0.8	111	26	Y	5	no	3	948
P5	76–80	11	26	−1.3	134	26	N	6	no	4	850
P6	71–75	15	24^*^	−2.0	111	24	N	9	no	4	650
P7	71–75	12	26	−2.5	115	26	Y	10	no	3	500
P8	51–55	16	28	−1.3	128	28	N	4	yes	2	513
P9	71–75	11	22^*^	−3.0	93	22	Y	23	no	4	1053
P10	61–65	17	25^*^	−0.2	123	25	N	10	no	2	900
P11	56–60	17	28	−0.2	123	28	Y	5	yes	2	1150
P12	56–60	17	24^*^	−1.8	111	24	N	7	yes	2	800
P13	76–80	11	21^*^	−2.4	115	21	Y	8	no	4	1200
P14	51–55	15	27	−0.2	111	27	N	6	no	1	925
P15	51–55	11	28	−1.3	123	28	N	6	no	2	2182
C1	61–65	11	27	−0.2	115	27					
C2	71–75	14	26	−1.3	115	26					
C3	65–70	12	29	−0.2	123	29					
C4	71–75	10	29	−0.2	115	29					
C5	51–55	13	27	0.8	123	27					
C6	71–75	10	27	−0.2	123	27					
C7	71–75	12	28	0.8	123	28					
C8	61–65	15	27	0.8	123	27					
C9	65–70	13	30	0.8	123	30					
C10	71–75	9	30	0.8	108	30					
C11	51–55	13	29	−0.8	139	29					
C12	56–60	13	30	−0.2	134	30					
C13	56–60	22	30	−0.8	143	30					
C14	56–60	16	28	−0.8	128	28					
C15	56–60	11	29	−0.2	134	29					

a *Subjects: P1-P15, patients, C1-C15, control*.

b *MoCA values below 26 indicate*

* *cognitive impairment. Gender: f, female, m, male, MWT, Mehrfachwahl-Wortschatz-Intelligenztest, MoCA, Montreal Cognitive Assessment, MDS UPDRS P1, Movement Disorder Society-sponsored revision of the Unified Parkinson's Disease Rating Scale Part 1 including 13 ratings ranging from 0 (normal) to 4 (severe) with a highest possible total score of 52, DBS, deep brain stimulation, H-Y stage, Hoehn-Yahr stage*.

The study was approved by the Ethics Committees of the Cantons of Thurgau and Bern, Switzerland; all participants gave written informed consent prior to participation. The study was conducted in accordance with the latest version of the Declaration of Helsinki.

### Embedded Face Paradigm

**Figure 1:**
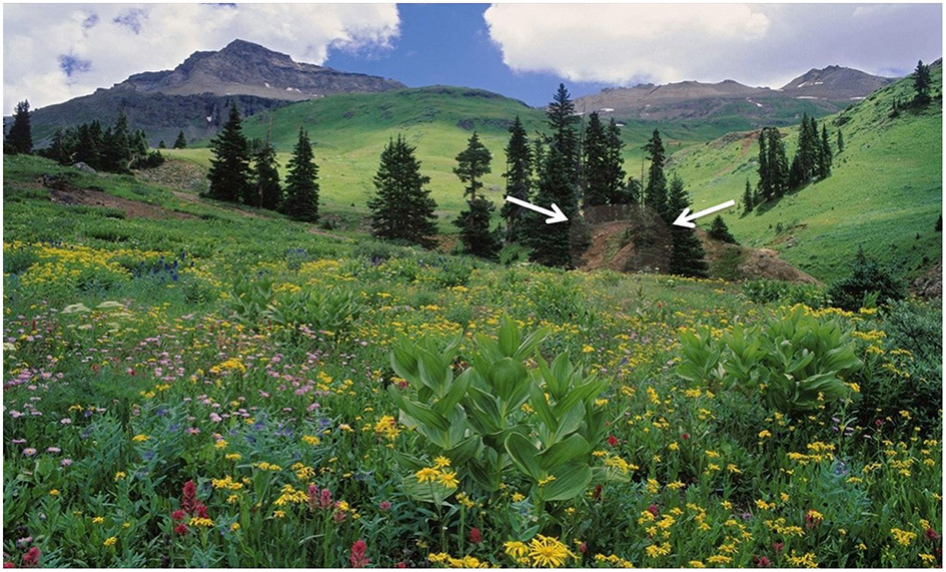
Stimulus material. Photograph with a human face embedded, marked by two white arrows.

The stimulus material consisted of 47 naturalistic color photographs of everyday scenes (see Figure 1). In 83% of the photographs, a human or animal face was embedded, the remaining 17% of the photographs did not contain a face. The “distinguishability” of the embedded faces was modified by decreasing the transparency of the face, resulting in a spectrum of images in which the difficulty to detect the embedded face varied from easy to very difficult.

### Experiment

The task was tablet-based and administered via a “13” Wacom Cintix 13HD (Wacom Co., Ltd., Otone, Saitama, Japan). The 47 images were presented for a maximum of 20 s. The participants were instructed to look at the images and, if they found an embedded face, to point with their finger on the face to identify it. In the case they would not find an embedded face, they had to state this. The experimenter noted all answers. Each subject performed a training run with three additional pictures (two with hidden faces and one without), in order to ensure that the participants understood the instructions. All PD patients underwent the assessment in their “on” state.

### Answer Types

The following types of answers were possible (see Table 2) and observed in our participants: Type 1 answer: A face was embedded in the image, but the participant did not find it. Type 2 answer: the participant correctly found the embedded face; Type 3 answer: The participant did not perceive the embedded face, but perceived a face that was *not* actually there at another location; Type 4 answer: No face was embedded in the image but a face was reported; Type 5 answer: No face was embedded in the image, and none was reported. Consequently, answers of type 2 and 5 are correct answers. Answers of type 1, 3, and 4 are errors, and, in particular, answers of type 3 and 4 can be considered as pareidolias.

**Table 2:** Possible types of answers in the embedded faces paradigm.

**Type**	**Embedded face in image**	**face identified**	**Answer**
1	Yes	No	wrong
2	Yes	Yes	correct
3	Yes	no, but another face was indicated at another location in the photograph	Pareidolia
4	No	Yes	Pareidolia
5	No	No	correct

### Statistical Analysis

The statistical analyses were performed using R, Version 3.5.0 (R Foundation for Statistical Computing, Vienna, Austria).

We calculated the frequencies of the different types of answers in the PD and the control group. In a next step, we calculated contingency tables for the values, applied Pearson chi-square statistics, and performed Mosaic Plots using R; In Mosaic Plots, the size of the fields is proportional to the frequency of the respective answers. Linear regressions were performed on the data of type 3 and type 4 answers, i.e., pareidolias, since these were of particular interest. Finally, receiver operating characteristic (ROC) curves were analyzed, and their areas under the curve (AUC) were calculated, in order to evaluate the sensitivity and specificity of each answer type in predicting whether a participant was a PD patient or not. Finally, a logistic regression model, classifying all error types, was performed to improve the prediction.

## Results

### Frequencies of Answer Types

**Figure 2:**
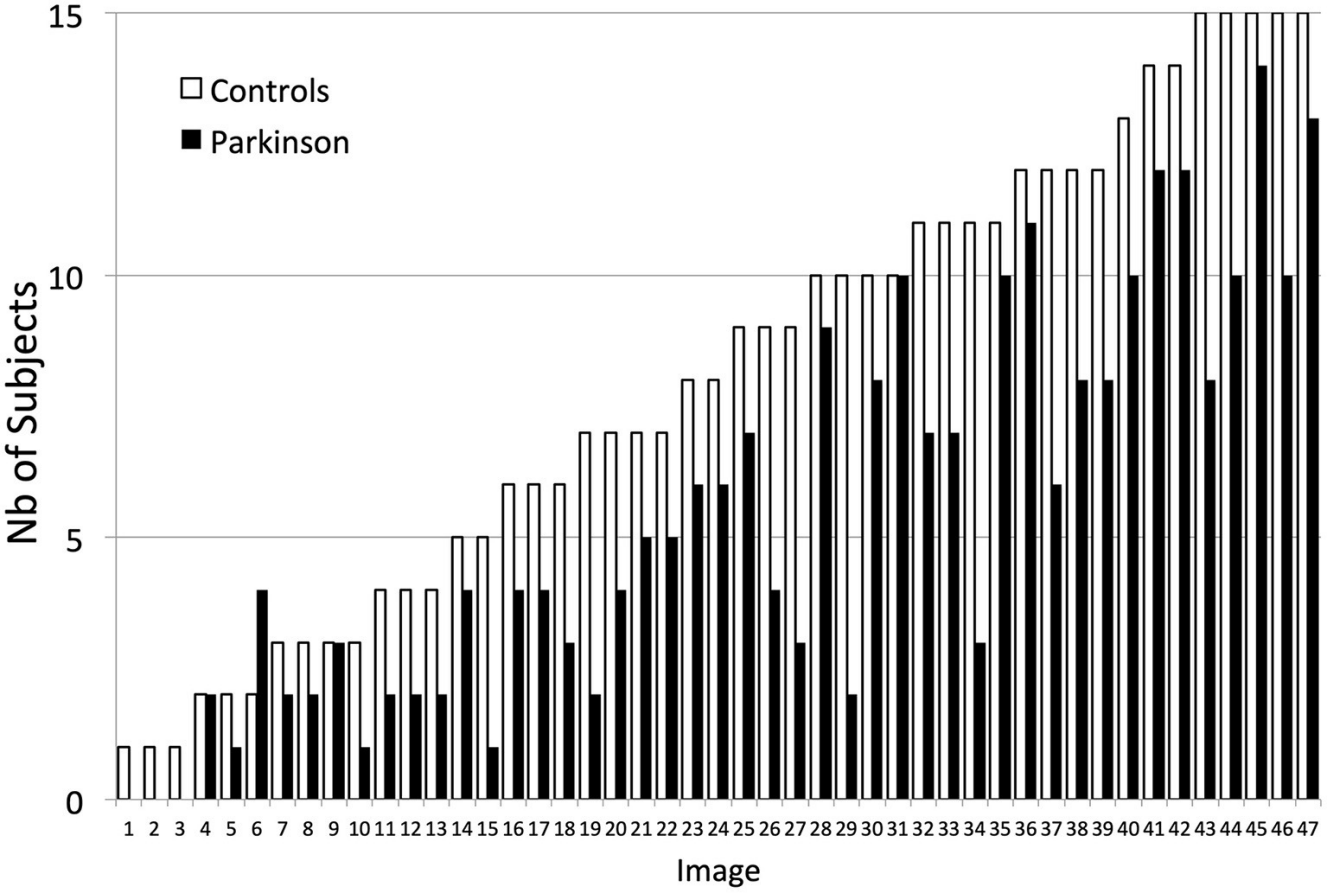
Performance (number of correct trials) in Controls and in PD patients across the 47 individual images. Results are presented in increasing order (i.e., from lowest to highest number of participants giving a correct answer to a given image).

Figure 2 presents descriptive statistics concerning performance (number of correct trials) of the participants for each image; the performance was sorted in increasing order, i.e., images with lower to higher numbers of correct answers. Our manipulation, varying the distinguishability of the embedded faces, resulted indeed in a linear increase of performance in controls over images (i.e., from images in which very few participants gave a correct answer, to images in which almost all participants responded correctly). For PD patients, the performance was in general lower.

**Figure 3:**
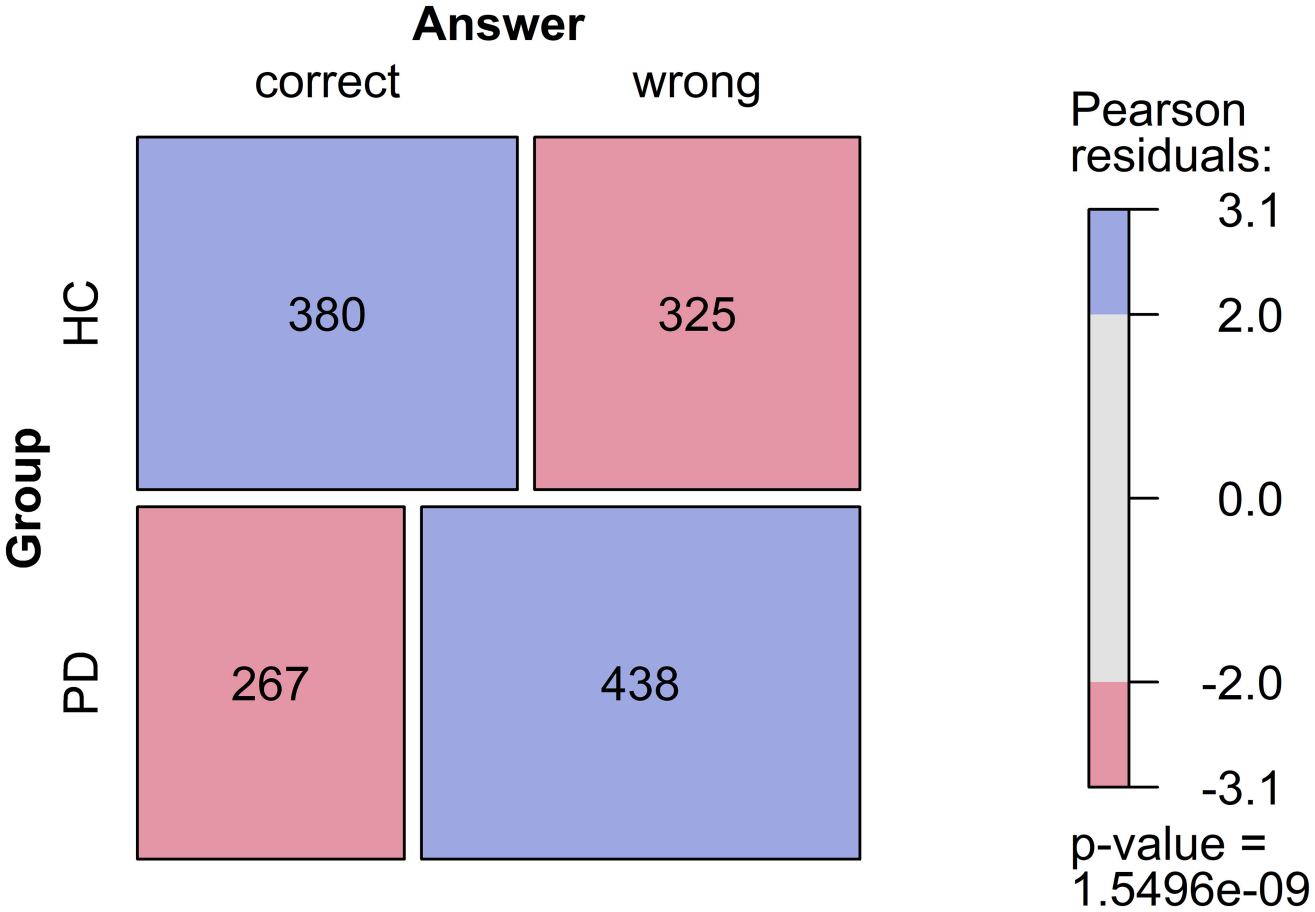
Mosaic plot of number of correct and wrong answers in Parkinson's disease patients and controls. The size of the fields corresponds proportionally to the frequencies, according to the contingency table. An implemented Chi-square test assesses whether the null hypothesis (H0) is true, i.e., that the variables are independent. Colors indicate which cells contribute to the significance of the Chi-square test result. Blue, there are more observations in this field than it would be expected under H0. Red, there are fewer observations in this field than it would be expected under H0.

Based on the frequencies of the possible answer types, we generated contingency tables, which served as a basis for Mosaic Plots with integrated Pearson chi-square statistics. The first Mosaic Plot (Figure 3) shows the results of the correct/wrong performance. PD patients made significantly more errors than controls. Controls produced 54% of correct answers, whereas PD patients produced 38% correct answers. This difference was statistically significant (*p* = <0.001).

**Figure 4:**
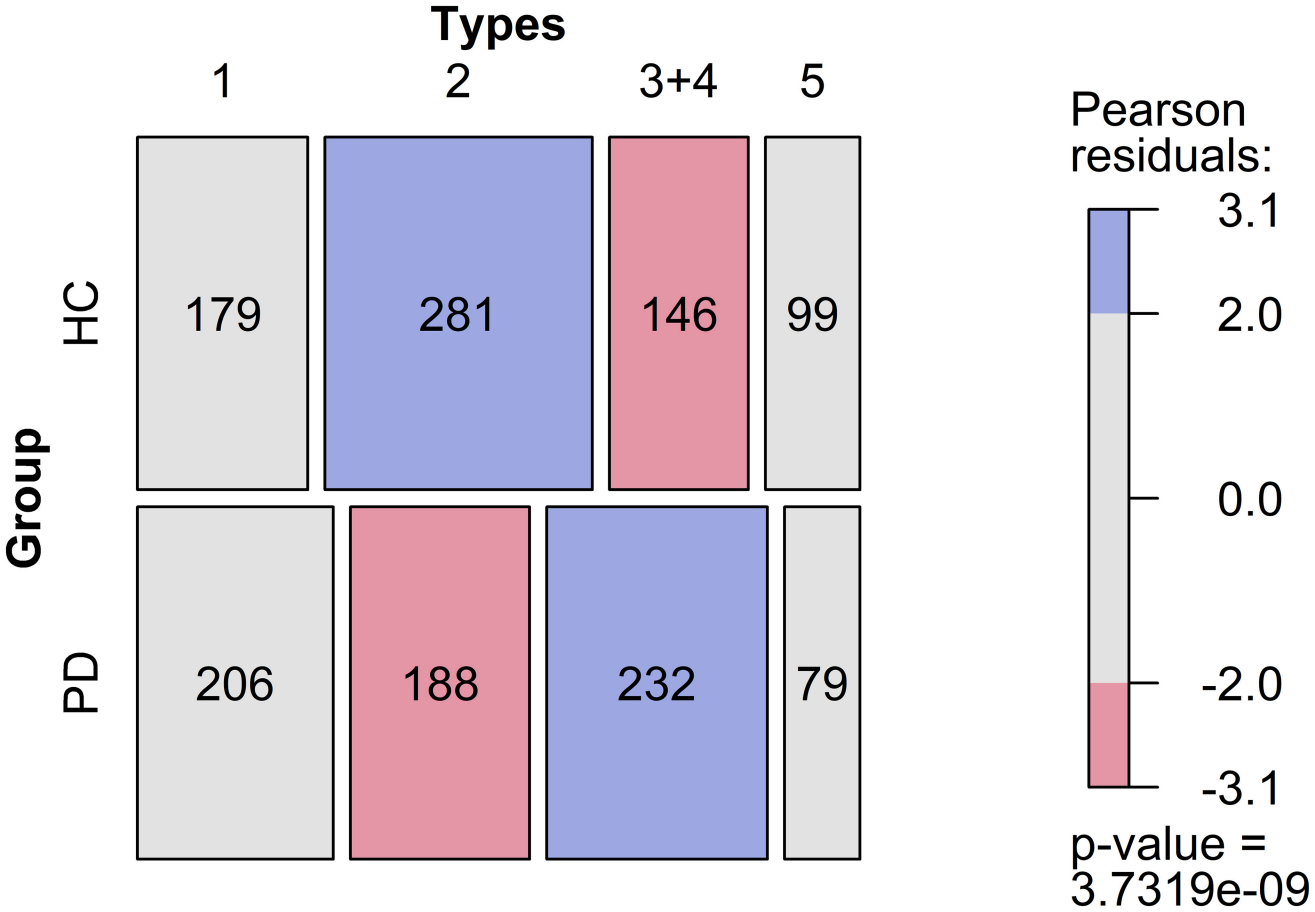
Frequency of different types of errors in controls and patients with Parkinson's disease. 1, embedded face not found, 2, face found, 3+4, face found where there was no face, 5, correctly identified that there was no face.

A detailed analysis of frequency of the different types of answers is shown in Figure 4. Independence between subject groups and answer types could be clearly rejected (*p* = <0.001). The frequencies of type 1 and 5 answers were not significantly different between the two groups. The frequency of type 2 answers (faces correctly identified) in the PD group was significantly different from the one in the control group. Type 3 (face not correctly identified, but pareidolia) and type 4 (pareidolia) answers, which reflect positive misperceptions and face pareidolia, were significantly more frequent in the Parkinson group than in the control group.

### Linear Regression Analysis of Answer Types

For linear regression analysis, the R package regr0 was used (30). To select an optimal regression model by means of the Akaike Information Criterion (AIC), the function step() was subsequently used.

#### Answer Type 3 (Embedded Face Not Identified, Face Pareidolia in Another Region)

A multiple linear regression was calculated to predict the frequency of answers of type 3 based on gender, age, presence of hallucinations, Stroop errors, MWT score, MoCA score, presence of DBS, and MDS P1 score in PD patients (Table 3). After using the function step(), a significant regression equation was found, based on MoCA [*F*_(1, 13)_ = 24.4, *p* = <0.001]. MoCA, as the only remaining variable in the regression explained 65.2% of the variance. The final predictive model was thus: *Type 3 errors* = 78.53–(2.59^*^MoCA).

**Table 3:** Output of the linear regression to predict answer type 3.

	**Coef**	**Df**	**ciLow**	**ciHigh**	**R2.x**	**Signif**	***p*-value**	***p*.symb**
(intercept)	78.529668	1	49.205277	107.854060				
**MoCA**	−2.589005	1	−3.721286	−1.456725	0	−2.29	**0**	***

*St.dev.error: 4.58 on 13 degrees of freedom*.

*Multiple R^∧^2: 0.652; Adjusted R-squared: 0.626; AIC: 47.51*.

*F-statistic: 24.4 on 1 and 13 d.f*.

Type 3: embedded face not found but another face reported (pareidolia), MoCA, Montreal Cognitive Assessment; coef, estimated value of the coefficient; df, degrees of freedom; ciLow and ciHigh, low and high boundary of the confidence interval; R2.x, coefficient of determination for regressing the explanatory variable in question on the other terms in the model (for collinearity diagnostics), signif, t-value/critical value, p value, whether the coefficient could be zero, p.symb, achieved significance level (***0.001, **0.01, *0.05, ^.^0.1).

#### Answer Type 4 (Classic Face Pareidolia)

Another multiple linear regression was calculated to predict the frequency of answers of type 4, based on gender, age, presence of hallucinations, Stroop errors, MWT score, MoCA score, presence of DBS, and MDS P1 score in PD patients (Table 4). After using the function step(), a significant regression equation was found, based on gender, age, presence of hallucinations, MWT score, MoCA score, and presence DBS [*F* (6,8) = 4.77, *p* = 0.0234], which explained 78.1% of the variance. Of all variables gender, age, hallucinations, and MWT were significant predictors of type 4 errors. MoCA and DBS were not significant. The final model was thus: *Type 4 errors* = *3.08*−*1.94 (female gender)* + *1.70 (male gender)*−*1.52 (hallucinating)* + *1.33 (not hallucinating)* + (0.15^*^age) + *1.24 (DBS)-*−0.31 (no DBS)—(0.15^*^MWT) + (0.34^*^MoCA).

**Table 4:** Output of the linear regression to predict answer type 4.

	**factor levels**	**Coef**	**df**	**ciLow**	**ciHigh**	**R2.x**	**signif**	***p*-value**	***p*.symb**
(intercept)		3.0773104	1	−14.63028202	20.78490289				
Gender	F/m	−1.94/1.70	1			0.362	1.68	0.005	**
Age		0.1492367	1	0.01904938	0.27942411	0.594	1.15	0.030	*
visual hallucination	Y/n	−1.52/1.33	1			0.331	1.35	0.015	*
MWT		−0.1505812	1	−0.26067308	−0.04048925	0.540	−1.37	0.014	*
MoCA		0.3416601	1	−0.31034478	0.99366489	0.656	0.52	0.261	
DBS	Y/n	1.24/−0.31	1			0.247	0.62	0.188	

*St.dev.error: 1.45 on 8 degrees of freedom*.

*Multiple R^∧^2: 0.781; Adjusted R-squared: 0.617; AIC:15.71*.

*F-statistic: 4.77 on 6 and 8 d.f., p-value: 0.0234*.

Type 4: no face embedded but face reported (pareidolia), factor levels: f, female; m, male; y, yes; n, no; MWT, Mehrfachwahl-Wortschatz-Intelligenztest; MoCA, Montreal Cognitive Assessment; DBS, deep brain stimulation; coef, estimated value of the coefficient; df, degrees of freedom; ciLow and ciHigh, low and high boundary of the confidence interval; R2.x, coefficient of determination for regressing the explanatory variable in question on the other terms in the model (for collinearity diagnostics), signif, t-value/critical value, p-value, whether the coefficient could be zero, p.symb, achieved significance level (***0.001, **0.01, *0.05, ^.^0.1).

### Receiver Operating Characteristic Curves (ROC) to Predict PD

**Figure 5:**
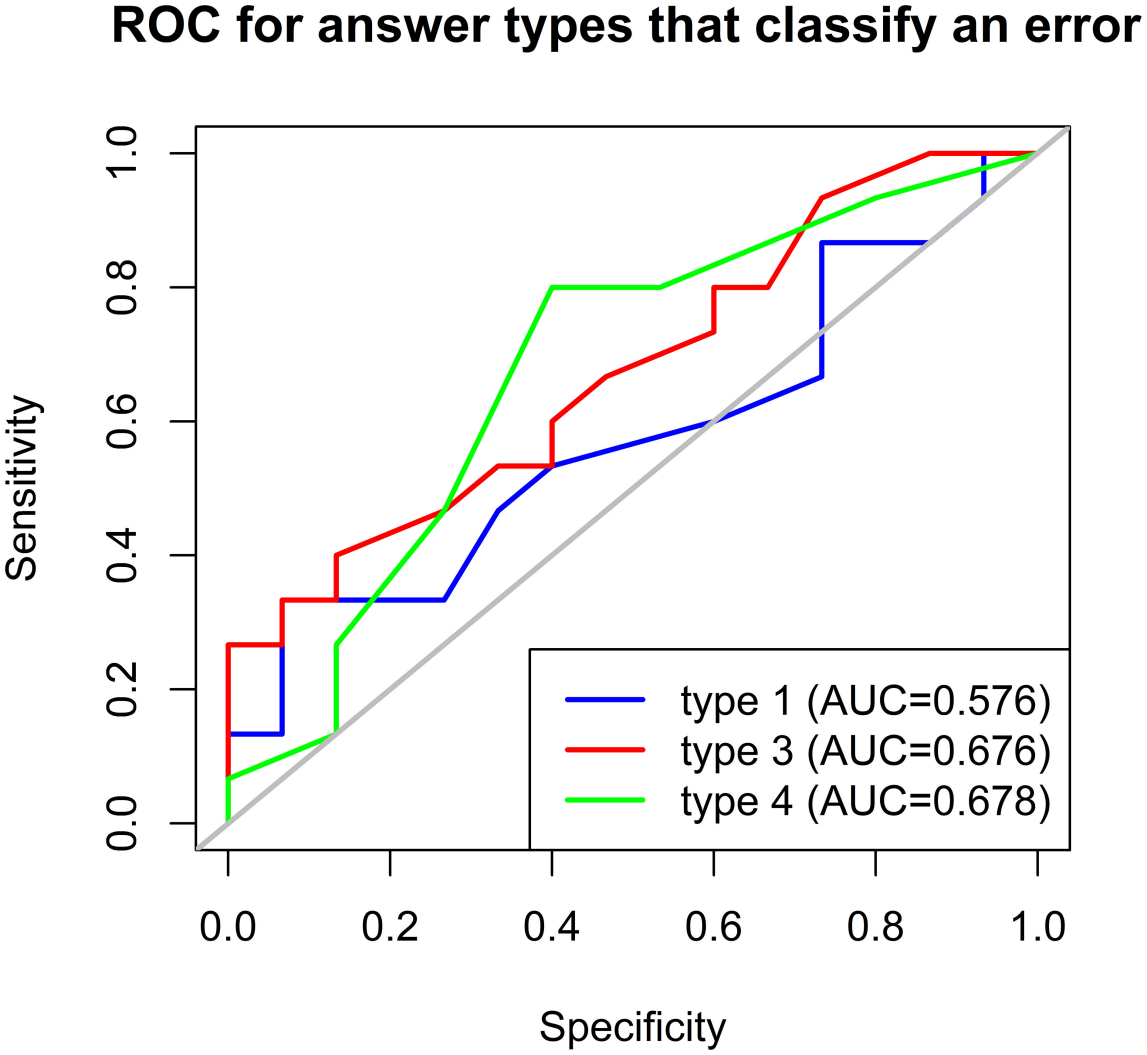
Receiver operating characteristic (ROC) curves to predict whether participants were PD patients based on answer types 1, 3, and 4 in the embedded faces paradigm. AUC, area under the curve.

To evaluate the sensitivity and specificity of the new paradigm in differentiating between PD and HC, receiver operating characteristic (ROC) curves were analyzed and their areas under the curve (AUC) were calculated. To this end, the answer types reflecting an error (i.e., answer types 1, 3, and 4) were considered (Figure 5). These answer types were found to perform moderately well-when considered individually (*AUC* for type 1 = 0.576, *AUC* for type 3 = 0.676, and *AUC* for type 4 = 0.678), but acceptably well-when summed up (*AUC* = 0.729).

**Figure 6:**
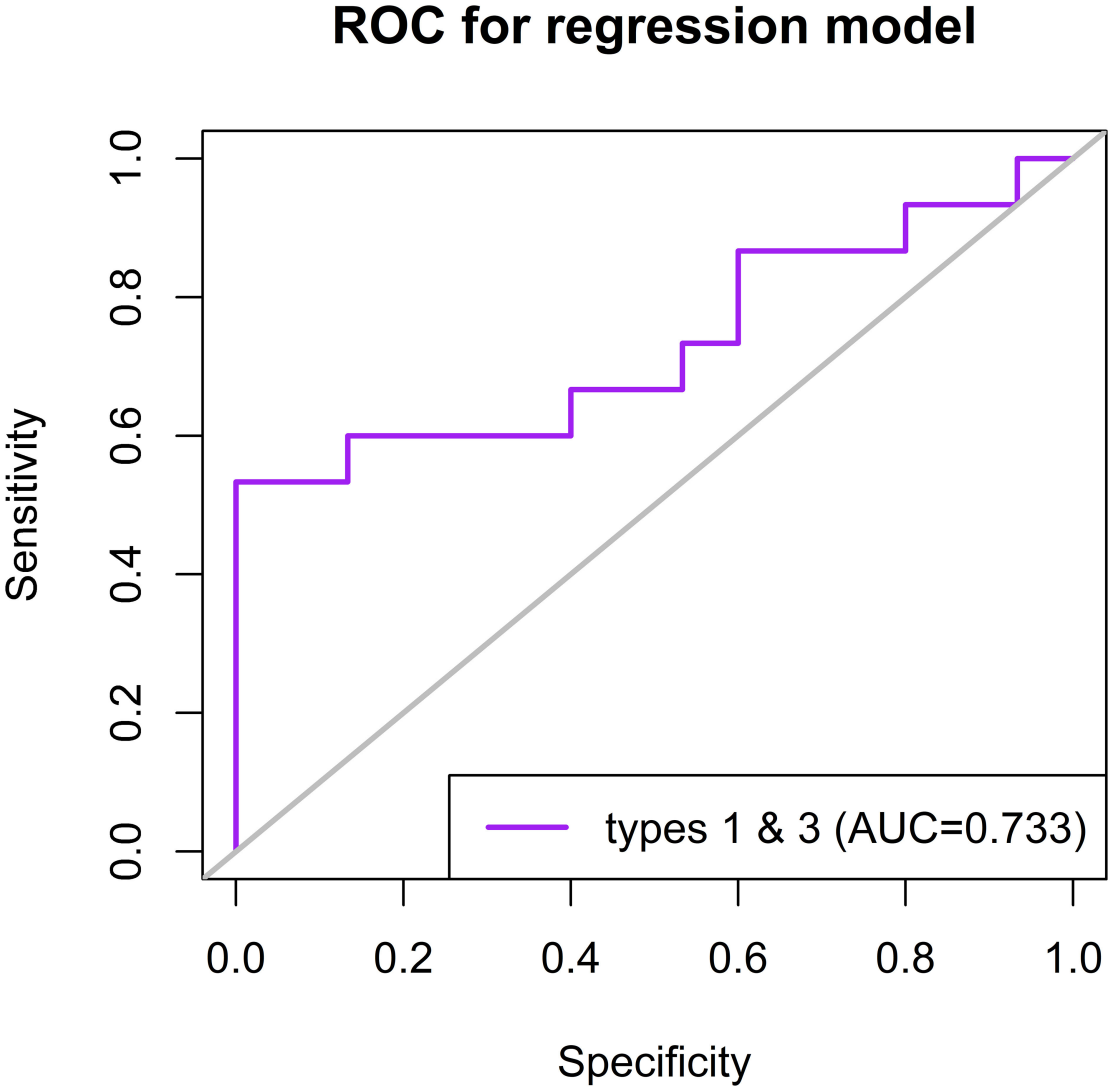
Receiver operating characteristic (ROC) curve to predict whether participants were PD patients based on the logistic regression model with answer types 1 and 3 in the embedded faces paradigm. AUC, area under the curve.

Finally, a logistic regression model including the errors was conducted. By using a backward model selection algorithm based on the Akaike information criterion (AIC), a significant regression equation was found. The predictor variables, number of type 1 answers [95% *CI* (−0.02, 0.30), *p* = 0.073], and number of type 3 answers [95% *CI* (0.01–0.31), *p* = 0.016] were found to contribute to the model in the logistic regression analysis (Table 5). This model was found to perform acceptably well (*AUC* = 0.733) in predicting whether participants were PD patients (Figure 6).

**Table 5:** Output of the logistic regression to predict whether participants were PD patients.

	**Coef**	**df**	**ciLow**	**ciHigh**	**R2.x**	**Signif**	**p.value**	**p.symb**
(intercept)	−3.32006664	1	−6.43251337	−0.20761991				
answer type 1	0.13935312	1	−0.02441110	0.30311736	0.232	0.91	0.073	^.^
answer type 3	0.16209735	1	0.01419535	0.30999936	0.232	1.23	0.016	*
	**deviance**	**df**	**p.value**					
Model	6.61424090	2	0.0366					
Residual	34.97458993	27	^.^					
Null	41.58883083	29	^.^					

*Distribution: binomial. Dispersion parameter: fixed at 1*.

*AIC: 3.00 40.97*.

Type 1, embedded face not found; type 3, embedded face not found but pareidolia; coef, estimated value of the coefficient; df, degrees of freedom; ciLow and ciHigh, low and high boundary of the confidence interval; R2.x, coefficient of determination for regressing the explanatory variable in question on the other terms in the model (for collinearity diagnostics), signif, t-value/critical value, p-value, whether the coefficient could be zero, p.symb, achieved significance level (***0.001, **0.01, *0.05, .0.1).

## Discussion

The aim of the study was to concurrently examine face perception and pareidolia production in PD patients. To this end, we developed a new paradigm using embedded faces in real scenic photographs.

Compared to the control group, PD patients found significantly less embedded faces, suggesting a perception deficit for this category of stimuli. This finding confirms the extant literature concerning face perception in PD patients. Indeed, it is known (31–35) that facial recognition and perception are dependent upon visuo-spatial processing resources, and PD patients were found to show visuo-perceptual disorders and difficulties in visual tracking tests (36, 37), some of which may be attributed to deficits in visuo-spatial memory (38).

More importantly, we found two different types of pareidolia production that were significantly more frequent in the PD group: (1) PD patients reported more faces than HC in images that actually did not contain any embedded face; (2) in images that actually contained embedded faces, PD patients more frequently reported faces within regions that did not contain any, but failed to report the actual embedded faces. This second type of response suggests that, at the same time, face perception is impaired and face pareidolia is more frequently produced.

The literature suggests that patients with PD (20) and other neurodegenerative diseases, such as Lewy body dementia (19, 21) and Alzheimer's disease (19), show more frequently pareidolia than HC. Furthermore, Sasai-Sakuma et al. (39) studied the production of pareidolia in 202 patients with idiopathic rapid eye movement sleep behavior disorder (iRBD). They found that 53.5% of iRBD patients exhibited significantly more pareidolic responses than the controls (21.7%). Furthermore, they found that the number of pareidolic responses was associated with cognitive decline in these patients. Multiple and recurrent visual complaints such as double vision, misjudging objects when walking, words moving whilst reading, and freezing in narrow spaces are common in PD, and also seem to be risk factors for minor forms of hallucinations (40).

The predictors of pareidolia production we found were based on the variables gender, age, presence of hallucinations, MWT score, MoCA score, and presence of DBS, which explained 78.1% of the variance. Significant predictors were gender, age, presence of hallucinations, and MWT score, a test for the evaluation of general intelligence. In the multiple linear regression to predict face pareidolia in images with embedded faces that were not detected (type 3 errors), we found that the variable MoCA score was the only remaining variable in the regression that significantly contributed to the model, explaining 65.2% of the variance. These results may suggest different mechanisms and predictors for these two types of pareidolia production. Furthermore, they support the idea that impaired cognition increases the risk of misperceptions in PD.

There are only a few studies concerning the physiological mechanisms subtending face pareidolia. Akdeniz et al. (41) studied the activation patterns in both a real-face and a face-pareidolia condition using fMRI in healthy subjects. They found that face-pareidolia triggers an interaction between top-down and bottom-up brain regions, including fusiform face area (FFA), frontal, and occipito-temporal regions. Furthermore, the right prefrontal cortex seems to play an important role both in processing real faces and face pareidolia, as did the FFA.

In a follow-up study, the same authors (42) analyzed event-related potentials (ERPs) elicited by actual faces and by face pareidolias in PD patients. They found that ERPs associated with both face and face-pareidolia processing are altered in PD patients, as reflected by early-stage neurophysiological activity in the temporo-parietal cortex.

In PD patients without cognitive impairment using resting state fMRI, Kajiyama and colleagues (43) found that PD patients who presented with pareidolia showed lower MMSE scores than the other PD patients who did not. Region of interest (ROI) analyses showed decreased functional connectivity between the prefrontal cortex and the face-recognition network in PD patients with pareidolia.

Uchiyama et al. (20) examined PD patients without dementia using FDG-PET. They found that PD patients produced a greater number of pareidolic illusions compared to the controls. Pareidolia were observed in all of the patients having visual hallucinations. This observation is in line with our results. Furthermore, they found a correlation between visual hallucinations and hypometabolism in the left parietal cortex. The authors suggest that posterior cortical dysfunction could be a common neural mechanism of pareidolia and visual hallucinations, and pareidolia could represent a subclinical form of hallucination, or a predisposition to visual hallucinations.

A study using visual exploration and pre-saccadic potentials (44) found that patients prone to pareidolia showed a significantly higher presaccadic potential on frontal electrodes suggesting a stronger frontal activation for pareidolic stimuli. Furthermore, by analyzing exploration behavior, they showed that PD patients need longer to convey attention to pareidolic stimuli. This is due to abnormal saccade generation in PD patients, which is proportional, on the one hand, to their visuo-perceptual deficits during early search, and, on the other hand, to time-independent alterations within the visual attentional network during late search.

Shine et al. (13) propose that visual misperceptions and hallucinations in PD may arise from disrupted processing within the attentional networks. They found in their fMRI study that patients who scored a high percentage of misperceptions and missed images were less able to activate frontal and parietal hubs of the Dorsal Attention Network (DAN). Furthermore, poor performance was significantly correlated with the degree of decreased activation in these hubs. Finally, patients with impaired performance displayed decreased resting state functional connectivity between hubs of the DAN and Ventral Attention Network (VAN). The VAN detects behaviourally relevant stimuli, irrespective of their positions in space. The DAN directs attention toward the spatial location of these behaviourally important stimuli. VAN plays thereby the role of a “circuit-breaker,” i.e., gives input to the DAN, in order to interrupt current activity and redirect visual attention (45). We can thus speculate that the results observed in our study rely on the disturbed interactions between these two networks evidenced by Shine et al. (13). In images in which a face is present, this may be detected as a behaviourally relevant stimulus by the VAN. However, due to the deficient communication with the DAN in PD patients, the DAN would not direct attention to the correct location, identifying a face at a location where actually none is present (such as in type 3 errors).

## Conclusions

Our study shows that PD patients identified significantly less embedded faces than age-matched HC, but produced significantly more face pareidolia than controls. Significant predictors for face pareidolia production in PD patients were gender, age, presence of hallucinations, and MWT score. Performance in the MoCA was a significant predictor for the performance in trials in which an embedded face was not detected but a face pareidolia was produced.

## Data Availability Statement

The original contributions presented in the study are included in the article/Supplementary Material, further inquiries can be directed to the corresponding author/s.

## Ethics Statement

The studies involving human participants were reviewed and approved by the Ethics Committees of the Cantons of Thurgau and Bern, Switzerland (KEKTGPE2015/17). The patients/participants provided their written informed consent to participate in this study.

## Author Contributions

NG: formal analysis, methodology, visualization, writing draft, and review and editing. JM: conceptualization, formal analysis, project admin, supervision, and visualization. NH: data curation, investigation, resources, and review and editing. AB: investigation, resources, and review and editing. MO: investigation, resources, and review and editing. JI: investigation, and review and editing. PU: writing draft, review and editing. DC: methodology, visualization, writing draft, review and editing. RM: conceptualization, formal analysis, funding acquisition, methodology, validation, writing draft, review and editing. All authors contributed to the article and approved the submitted version.

## Conflict of Interest

The authors declare that the research was conducted in the absence of any commercial or financial relationships that could be construed as a potential conflict of interest.

## Publisher's Note

All claims expressed in this article are solely those of the authors and do not necessarily represent those of their affiliated organizations, or those of the publisher, the editors and the reviewers. Any product that may be evaluated in this article, or claim that may be made by its manufacturer, is not guaranteed or endorsed by the publisher.
